# Pathways to promote the performance of healthcare financial expenditure from an audit perspective: Evidence from Hunan Province

**DOI:** 10.1371/journal.pone.0341362

**Published:** 2026-01-27

**Authors:** Shengchun Ling, Xiaoqi Tang, Qiaojun Shi

**Affiliations:** School of Business, Hunan Agricultural University, Changsha, China; Central South University School of Architecture and Art, CHINA

## Abstract

Strengthening the audit supervision of healthcare financial expenditures and improving the performance of healthcare expenditures are critical to achieving the Healthy China strategy. This study uses data from 14 cities in Hunan Province from 2013 to 2022 and employs the DEA-Malmquist analysis method to analyze the existing problems and institutional incentives for healthcare financial expenditures in Hunan Province on the basis of estimating the performance of healthcare financial expenditures in each city. The results show that the overall performance level of healthcare financial expenditure in Hunan Province is low, and the efficiency of healthcare expenditure is trending downward. The majority of cities are in the state of increasing scale efficiency of healthcare expenditure, with insufficient scale of inputs. The main reason for the decline in efficiency is the low technological progress change index. The fundamental reasons are the shortcomings of the healthcare financial expenditure system, insufficient binding force, and inadequate supervision. Based on this, the article explores the audit mechanism of changes in expenditure performance, and then proposes specific ways for the audit to promote the improvement of healthcare financial expenditure performance, in order to give full play to the supervision and guarantee role of the audit in promoting healthcare financial expenditure performance.

## Introduction

Since the national government officially launched the deepening of medical and health system reforms in 2009, both central and local governments have increased fiscal investments in the medical and health system to achieve reform objectives. From the new medical reform in 2009–2022, Hunan Province, a pivotal region in central China, saw its local spending on medical and health services rise from 15.92 billion yuan to 82.061 billion yuan. The proportion of healthcare expenditure in the general budget of local finances has grown from 7.20% to 9.13% [1]. Healthcare investments by municipal governments have also continued to expand, effectively driving the rapid development of Hunan Province’s healthcare sector.

However, the expansion of fiscal capital investment has not been fully translated into corresponding improvements in service efficiency. As the entrusted manager of public resources, the government faces problems of information asymmetry and incentive incompatibility in the principal-agent relationship related to medical and health expenditures. It needs to ensure the performance of public entrusted responsibilities through effective supervision mechanisms. As a vital component of the national governance system, government auditing primarily enhances governance effectiveness by exercising oversight, imposing constraints, and facilitating information disclosure [2]. Strengthening audit supervision in fiscal expenditure and public service domains can reveal deviations in fund utilisation, rectify inefficient resource allocation practices, and drive institutional improvements and management optimization [3]. This process standardises expenditure behaviour while ultimately enhancing the efficiency of fund utilisation.

The problem of inefficient use of funds in the medical and health industry is common. Taking Hunan Province as an example, the 2022 audit report by the Provincial Audit Office revealed that during special audits of medical insurance funds and the ‘three-sector medical reform’ initiative, 71 medical institutions across four sampled cities were found to have engaged in unauthorised charging practices. Five institutions were implicated in irregular procurement of pharmaceuticals and medical consumables amounting to 581 million yuan, alongside issues such as defrauding medical insurance funds, unauthorised mark-ups on sales, and defaulting on supplier payments [4]. These issues not only result in the loss and waste of public funds but also constrain improvements in the performance of healthcare expenditure [5]. As social problems such as population aging intensify, the public’s demand for high-quality medical services continues to grow. The contradiction between the improvement of overall medical and health service levels and the increase in medical and health financial burdens has become increasingly prominent.

Based on this, the National Audit Office issued the “14th Five-Year Plan for the Development of National Audit Work” in 2021, which emphasizes the need to establish a centralized, unified, comprehensive, and efficient audit supervision system, and to organize audits on the progress of the health system construction and reforms, to promote the implementation of the Healthy China strategy. Therefore, this study examines the performance of healthcare fiscal expenditures in Hunan Province as an example, systematically analyzing the use effect, spatiotemporal evolution characteristics, and regional differences of healthcare fiscal funds in Hunan Province, and deeply analyzes the mechanism of government auditing in improving the performance of healthcare fiscal expenditures. By systematically examining the internal logic of auditing to exert supervisory effectiveness through revealing problems, promoting rectification, and promoting system improvement, and on this basis, policy recommendations for specific problems in Hunan Province’s healthcare financial expenditures are constructed. It not only helps to improve the efficiency of the use of healthcare financial funds in Hunan Province and promotes the coordinated development of cities in the province, but also provides a reference for other provinces in the central region, which has important practical significance for promoting the sustainable development of healthcare undertakings.

## Literature review

Government provision of healthcare resources is finite. Enhancing the performance of healthcare expenditure is of paramount importance for the government to effectively fulfil its public responsibilities and meet the public’s growing demands for public health [6]. As the trustee, the government accepts the public’s mandate to use and manage fiscal funds reasonably and effectively in accordance with relevant national laws, regulations, and rules. On this basis, it provides healthcare services to the public. However, the multi-layered agency structure exacerbates information asymmetry, where agents may act contrary to the principal’s interests, causing resource allocation to deviate from its intended objectives [7]. In order to deal with the reduced efficiency caused by agency problems, effective institutional arrangements need to be constructed to constrain the behavior of agents. Government audit plays an indispensable role as a vital component of oversight mechanisms. Through independent supervision, it reduces information asymmetry, strengthens accountability mechanisms, and coordinates multi-level incentive structures, thereby enhancing the performance of fiscal fund utilisation [8,9].

Regarding research on the performance of healthcare financial expenditure and its influencing factors, the stochastic frontier analysis (SFA) method and the data envelopment analysis (DEA) method are mainly employed by scholars. Cheng and Liao [10] used heterogeneous stochastic frontier analysis and found that the efficiency of medical and health resources nationwide showed an increasing trend. Yu and Shi [11] used SFA to explain the problem of health efficiency changes from the perspective of cost constraints and expenditure uncertainty. The stochastic frontier method needs to pre-set the form of the production function and consider the random error term. When dealing with multiple outputs, the correlation between indicators will also have an impact on the reliability of the results. The DEA method and its extended model are widely used in public sector efficiency evaluation. Its advantage is that it does not need to preset the form of the production function and can handle multiple inputs and multiple outputs problems at the same time [12,13]. Carrillo and Jorge [14] applied the DEA model to evaluate the efficiency of regional healthcare systems in Spain and identify regions that make more effective use of healthcare inputs. Guo et al. [15] used the DEA and the Malmquist productivity index to evaluate and analyze the performance of healthcare expenditure in the context of the new healthcare reform and identified the causes of efficiency changes. On this basis, some studies have thoroughly explored the specific factors that affect the performance of healthcare fiscal expenditures, showing that per capita GDP, medical technology level, medical environment, education level, fiscal decentralization, and fiscal transparency all affect the performance of healthcare financial expenditure to varying degrees [16–20]. Some scholars have also studied the differences in healthcare financial expenditure. Halkos and Tzeremes [21] argue that the Greek government’s public medical management among regions is still poor, and there are still differences in the allocation of medical care resources between cities and rural areas. Most scholars’ research on China believes that there are obvious differences in the efficiency level of medical and health expenditures among provinces [22]. The efficiency is ranked from high to low in the eastern region, the central region, and the western region, and there are also large differences within regions [23,24]. However, research also shows that the differences within regions are constantly shrinking [25]. Existing research has laid a methodological foundation for the performance evaluation of healthcare financial expenditures. However, most studies focus on China’s national level or the developed eastern regions, and a systematic evaluation of the performance levels and dynamic changes of healthcare financial expenditures in central provinces is still insufficient.

At present, research on government audits primarily focuses on two areas. Some studies explore the value and role of government audits. The objective of government audits is to oversee the fulfillment of public fiduciary responsibilities, with a particular emphasis on the standardization of power exercise, the rational use of fiscal funds, the optimal allocation of resources, and the quality of public services [26]. Through systematic management and control mechanisms, government audits ensure compliance and efficiency [27–30]. Other literature examines the role of government audits in ensuring the lawful and compliant use of public resources and enhancing economic efficiency. It is argued that government audits, by monitoring the exercise of power and the use of fiscal funds, can enhance fiscal transparency, curb government corruption, reduce wasteful spending, and improve the efficiency of public resource utilization [31–35]. A few scholars have explored the pathways through which government audits influence public expenditure. For instance, scholars Zhang et al. [36] argue that national audits can positively impact the efficiency of public fiscal expenditure by leveraging their disclosure, resistance, and preventive functions. Research by Lu et al. [37] also indicates that national audits can effectively address corruption in government health expenditure, with greater audit intensity contributing to higher levels of government health expenditure. However, few scholars have studied the path to improving the performance of healthcare financial expenditure from an audit perspective, and research on specific provinces remains particularly limited. As a typical representative of the central region, Hunan Province’s financial investment in medical and healthcare continues to grow, but performance improvement faces many challenges. Therefore, this article starts with the performance of healthcare financial expenditures. On the basis of theoretical analysis, it uses data envelopment analysis to evaluate the performance of Hunan Province’s medical and health financial expenditures, deeply explores the institutional roots that affect its performance, and reveals the role of audit supervision in performance improvement. On this basis, it provides a theoretical basis and policy suggestions for auditing to promote the performance improvement of Hunan Province’s medical and health financial expenditures, and provides a reference for other provinces in the central region.

## Performance evaluation methods and results analysis

### Evaluation methods

#### DEA-BCC model.

The DEA model is one of the most commonly used methods in academia for evaluating the performance of healthcare expenditures [38]. The Charne, Cooper, Rhodes (CCR) model and the Banker, Charnes, Cooper (BCC) model are commonly used basic models in DEA, which can be used to compare the production efficiency of decision-making units (DMU) at the same time point. Since the premise of the CCR model is that returns to scale remain unchanged, and there are economies of scale in medical and health expenditures, the BBC model can be used to more effectively calculate the economies of scale of DMUs [39,40]. This study employs the DEA model to conduct a performance audit and evaluation of healthcare expenditures, with the 14 cities of Hunan Province serving as DMUs.

#### Malmquist productivity index.

The ordinary DEA model measures the static technical efficiency based on production technology at a certain time, but production is a long-term, continuous process, production technology is constantly developing and progressing, and technological progress plays a key role in improving productivity [41]. The Malmquist Productivity Index is based on this, a dynamic analysis of total factor productivity change (TFPCH) across different periods for DMUs, reflecting changes in productivity from period t to t + 1. Its average level indicates the contribution of expenditure efficiency to output growth [42,43]. The TFPCH can be broken down into technical efficiency progress change (EFFCH) and technical progress change (TECHCH), where EFFCH can further be decomposed into changes in pure technical efficiency change (PECH) and changes in scale efficiency change (SECH). If the indices are greater than 1, it indicates an increase in efficiency; if they equal 1, it indicates no change in efficiency; and if they are less than 1, it means a decrease in efficiency.

### Selection of indicators and data sources

For the selection of input variables, this study refers to the research of Gong et al. [44] and Yang et al. [45], directly using the indicator of per capita local government expenditure on healthcare as the input variable. Healthcare expenditure is the most direct measure of public health fiscal investment in public health. Output indicators are selected based on the three key health resources that best represent Hunan Province’s capacity to provide healthcare services: healthcare facility count, healthcare facility bed count, and healthcare professional count [46–48]. All data used were sourced from the Hunan Province Statistical Yearbook (2013–2022) and the National Economic and Social Development Statistical Bulletins published by the Hunan Provincial Bureau of Statistics for each city.

### Evaluation results and related analysis

#### Static analysis based on the DEA-BCC model.

Based on the selection of input-output indicators mentioned above, the Deap2.1 software was used to calculate the performance of medical and health financial expenditures of 14 cities in Hunan Province in 2022 from the perspectives of variable returns to scale and output. Table 1 summarises the static efficiency and composition of medical and health financial expenditures in each city in Hunan Province in 2022. Technical efficiency (TE) comprehensively reflects the overall efficiency level of medical resource allocation and is decomposed into two dimensions: pure technical efficiency (PTE) and scale efficiency (SE). Among them, PTE measures the utilization efficiency of fiscal funds in allocating health resources, reflecting the technical and management capabilities of various cities in converting fiscal expenditures into health resources under the same level of fiscal investment. SE reflects the degree of deviation between the current per capita fiscal expenditure scale and the optimal scale of output health resources. The calculation results show that only Changsha achieves a PTE, SE, and TE of 1, indicating DEA effectiveness. Xiangtan and Shaoyang both achieved a PTE of 1, but their SE was not 1, indicating DEA weak effectiveness. The remaining 11 cities were all deemed DEA ineffective. From the overall mean values, the TE, PTE, and SE means for the 14 cities were 0.685, 0.878, and 0.771, respectively, none of which reached DEA efficiency, indicating that the performance of healthcare financial expenditures in Hunan Province remains insufficient. Existing inter-provincial research has shown that the TE of healthcare financial expenditures in Hunan Province ranks sixth in the country, higher than most central and western provinces. However, this relative advantage at the inter-provincial level masks the significant differences between cities within the province. The TE of Changsha is 1.0, while the city with the lowest efficiency is only about 0.65. The largest gap within the province reaches 0.35, which significantly exceeds the TE difference of 0.15 among the six provinces in central China [49]. From the perspective of returns to scale (RTS), this indicator reflects the marginal effect of increasing per capita medical and health expenditures on health resource output. Changsha’s RTS remains unchanged. However, Shaoyang and Yongzhou, as relatively underdeveloped regions with lower per capita GDP in Hunan Province, show diminishing RTS and excessive investment. This breaks the cognitive misunderstanding of simply linking the level of economic development to input demand, indicating that the investment scale of some underdeveloped areas has exceeded the optimal level relative to their actual needs. The remaining 11 cities show increasing RTS, indicating to a certain extent that it is meaningful to increase medical and health fiscal expenditure. The above findings provide evidence support for government audits to curb unreasonable fiscal expenditure and inefficiency, reasonably adjust the scale of medical and health investment in different cities and prefectures, and improve financial expenditure performance.

**Table 1:** Performance and composition of Hunan Province’s healthcare expenditure in 2022.

Cities	TE	PTE	SE	RTS
Changsha	1.000	1.000	1.000	–
Zhuzhou	0.586	0.980	0.598	irs
Xiangtan	0.469	1.000	0.469	irs
Hengyang	0.813	0.942	0.863	irs
Shaoyang	0.977	1.000	0.977	drs
Yueyang	0.698	0.856	0.815	irs
Changde	0.814	0.819	0.993	irs
Zhangjiajie	0.184	0.728	0.253	irs
Yiyang	0.603	0.829	0.727	irs
Chenzhou	0.707	0.861	0.821	irs
Yongzhou	0.823	0.832	0.989	drs
Huaihua	0.759	0.841	0.902	irs
Loudi	0.829	0.974	0.852	irs
Xiangxi	0.335	0.623	0.537	irs
Mean	0.685	0.878	0.771	

#### Dynamic analysis based on the DEA-Malmquist method.

The Malmquist index is a dynamic efficiency analysis method grounded in the DEA model. This paper employs DEAP2.1 for computing the variations and component values of the Malmquist index in Hunan Province from 2013 to 2022.

**Fig 1:**
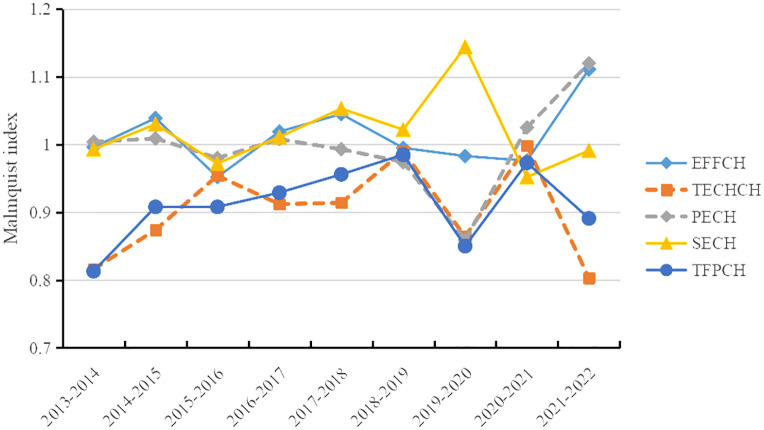
Changes in the malmquist index of healthcare financial expenditure performance in Hunan Province from 2013 to 2022.

Table 2 shows that the TFPCH values for Hunan Province from 2013 to 2022 were below 1, indicating varying degrees of negative growth in the performance of healthcare financial expenditure, exhibiting a trend of diminishing marginal utility. The TFPCH can be decomposed into TECHCH and EFFCH. The TECHCH reflect the movement of the production technology frontier in the medical and health field, and reflect the role of technological innovation, management innovation, and institutional innovation in the medical and health field in promoting the efficiency of health resource allocation. The EFFCH can be further decomposed into PECH and SECH. Among them, the PECH measures the improvement in management efficiency of fiscal funds allocation of health resources. The SECH reflects the dynamic process of adjusting the input scale to the optimal scale. As shown in Fig 1, the index value of TECHCH is the lowest in comparison. It rose steadily from 2013 to 2019, but after 2019, the value of TECHCH fluctuated significantly. Overall, the relatively low index value of TECHCH has influenced the trend in healthcare financial expenditure performance, leading to the TFPCH for healthcare fiscal expenditure in Hunan Province failing to reach an effective state. Therefore, technological progress is the most important factor constraining the growth of healthcare financial expenditure performance, and scientific performance improvement measures should be implemented to gradually enhance medical technology capabilities [50].

**Table 2:** Malmquist index and decomposition of healthcare financial expenditure performance in Hunan Province from 2013 to 2022.

Year	EFFCH	TECHCH	PECH	SECH	TFPCH
2013-2014	0.996	0.816	1.004	0.993	0.813
2014-2015	1.039	0.874	1.009	1.030	0.908
2015-2016	0.952	0.954	0.980	0.972	0.908
2016-2017	1.019	0.912	1.008	1.011	0.929
2017-2018	1.045	0.914	0.993	1.053	0.956
2018-2019	0.995	0.990	0.974	1.022	0.985
2019-2020	0.983	0.864	0.859	1.144	0.850
2020-2021	0.976	0.998	1.025	0.952	0.973
2021-2022	1.111	0.803	1.120	0.991	0.891
Mean	1.012	0.900	0.995	1.017	0.911

Table 3 delineates the alterations in the Malmquist index and its component indices for the average healthcare financial expenditure performance of cities and prefectures in Hunan Province from 2013 to 2022. As shown in Table 3, the three cities of Changsha, Loudi, and Zhuzhou rank highly in the TFPCH, with index levels exceeding 0.930. Among them, Changsha recorded the highest index value. Zhangjiajie, Xiangxi, and Shaoyang rank lower, indicating certain bottlenecks in the growth of healthcare financial expenditure performance. From the decomposition of the Malmquist index, the PECH of Changsha, Zhuzhou, Xiangtan, Shaoyang, and Loudi all exceed 1, while other cities still have room for improvement. Except for Hengyang and Shaoyang, all other cities reported SECH above 1, indicating that the scale efficiency of healthcare financial expenditures in Hunan Province has generally reached a relatively high level. Further analysis of the TECHCH reveals that this metric remains comparatively low across all cities relative to other indicators, with none achieving an effective state. This reaffirms that suboptimal technological progress constitutes the primary factor constraining the performance of healthcare financial expenditure. With the continuous expansion of financial investment in the medical and healthcare field, the problems that exist will become increasingly prominent. Therefore, it is of great significance to give full play to the ‘immune system’ function of government audits and establish a sound performance audit mechanism for financial expenditure on medical and healthcare to improve overall performance.

**Table 3:** Malmquist index of average healthcare expenditure performance and decomposition in various cities in Hunan Province.

Cities	EFFCH	TECHCH	PECH	SECH	TFPCH	Rank
Changsha	1.000	0.955	1.000	1.000	0.955	1
Zhuzhou	1.031	0.906	1.018	1.013	0.935	3
Xiangtan	1.025	0.901	1.009	1.016	0.924	4
Hengyang	0.995	0.910	0.996	0.999	0.905	8
Shaoyang	0.997	0.884	1.000	0.997	0.881	14
Yueyang	1.003	0.899	0.987	1.016	0.902	11
Changde	1.014	0.897	0.988	1.027	0.910	6
Zhangjiajie	1.003	0.898	0.979	1.024	0.901	12
Yiyang	1.012	0.895	0.983	1.029	0.906	7
Chenzhou	1.014	0.898	0.998	1.017	0.911	5
Yongzhou	1.014	0.892	0.995	1.019	0.904	9
Huaihua	1.010	0.896	0.989	1.022	0.904	9
Loudi	1.050	0.893	1.006	1.044	0.938	2
Xiangxi	0.999	0.883	0.982	1.018	0.882	13
Mean	1.012	0.900	0.995	1.017	0.911	

### Conclusions of the assessment of the performance

Using the DEA-Malmquist analysis method, a static and dynamic analysis of the performance of financial expenditure on medical and health care in 14 cities in Hunan Province was conducted from the perspective of input-output efficiency to explore the performance of financial expenditure on medical and health care by the governments of various cities in Hunan Province. The following conclusions can be drawn:

#### Low-performance levels and insufficient investment in healthcare expenditure.

Static analysis indicates that the TE of healthcare expenditure across Hunan’s cities in 2022 is relatively low, with significant differences between cities, and most cities exhibit increasing returns to scale. From the results of the dynamic analysis, the performance of medical and health financial expenditure in various cities exhibited a downward trend between 2013 and 2022. The above analysis results indicate that there is still great potential for improvement in the performance of medical and health financial expenditure in Hunan Province. Overall investment in medical and health resources is insufficient. Local authorities can improve the effectiveness of their health budgets by appropriately increasing the scale of investment while balancing the development between cities and reasonably allocating health resources between them.

#### Technological progress is the key factor constraining the improvement of total factor productivity.

According to the analysis of the Malmquist index, the TFPCH indices of all cities in Hunan Province are less than 1, indicating an overall decline in expenditure efficiency. The TECHCH has dropped significantly compared with other values, and the lag in technological progress has a greater impact on total factor productivity. Based on this, Hunan Province should be committed to scientific and technological innovation and make coordinated efforts in clinical technology, management efficiency, information construction, and other aspects. It should build a high-caliber medical talent pipeline, optimize the training system and incentive mechanisms for healthcare professionals, introduce modern management tools, accelerate the construction of digital infrastructure, and continuously enhance total factor productivity. These measures will ultimately improve the performance of healthcare financial expenditures across the entire province.

## Analysis of the reasons for the low-performance level and the role of auditing

### Reasons for the low-performance level of financial expenditure on medical and healthcare

#### Decision-making bias leads to efficiency losses.

Under the current GDP-led performance appraisal system, local government officials tend to allocate limited financial resources to areas and projects with small investment scales, short construction cycles, and quick results to pursue short-term political achievements, with the hope of achieving performance appraisal indicators and promotion in a relatively short period of time. However, healthcare, as an area of people’s livelihood with a long investment cycle, slow results, strong social spillover effects, and not a major promotion assessment indicator, is often marginalized in local government financial expenditure decision-making. As a province in the central region of China with significant economic development potential, Hunan Province faces dual pressures from the need to increase fiscal revenue and improve people’s livelihoods. Under these circumstances, local governments tend to prioritize fiscal expenditures on productive sectors such as infrastructure construction that can attract foreign investment and drive economic growth. For example, in underdeveloped regions like Xiangxi and Huaihua, despite insufficient medical resources, expenditures on economic development still account for a significantly higher proportion of the fiscal expenditure structure than those on healthcare. This decision-making orientation has led to low efficiency in Hunan Province’s healthcare expenditures due to insufficient investment scale, and this is also the fundamental cause of the slow technological progress identified in the previous Malmquist index analysis. Therefore, relying solely on substantial fiscal investments from local governments cannot effectively improve the performance of healthcare expenditures, which is closely related to the current decision-making system orientation.

#### Incomplete performance evaluation hinders technological progress.

Taking Hunan as an example, although the steady upswing in the financial expenditure into healthcare and medicine, the efficiency of fund utilization and management in the medical and healthcare sector has not improved at the same pace. Residents’ medical and healthcare expenditure levels remain problematic, which is closely related to the fact that an effective medical and healthcare expenditure performance evaluation system has not yet been established.

Specifically, the current practice of performance evaluation of healthcare fiscal expenditures in Hunan Province primarily faces the following issues. first, there is an excessive focus on project completion and compliance with funding usage, failing to reflect the social benefits and long-term value of healthcare expenditures. Second, the evaluation indicator system is not systematic and complete, and it lacks assessment of key indicators such as the operational efficiency of medical institutions, areas of the medical security system, and coverage of basic public health services. Additionally, there is a lack of systematic, quantifiable evaluation metrics for the diverse factors driving technological advancement, such as medical technology innovation, medical equipment updates, management process optimization, information system development, and service model reforms, making it difficult to effectively incentivize and guide technological progress and support the sustained improvement of total factor productivity. Thirdly, the mechanism for applying evaluation results is not yet fully established. The performance evaluation results of healthcare expenditures in Hunan Province have not been effectively integrated with substantive work such as fiscal budget arrangements and healthcare institution development plans. For example, in the construction of medical integration in the Changzhutan urban agglomeration, it is difficult to convert the performance evaluation results into a basis for decision-making on the optimal allocation of resources. There is a lack of follow-up tracking and continuous improvement of the evaluation results, making it difficult to effectively play the guiding role of performance evaluation in improving the efficiency of fund use. These problems make it difficult to supervise the efficiency and effectiveness of medical and health expenditures, affecting the actual effectiveness of the performance evaluation of medical and health financial expenditures and limiting the role of evaluation results in subsequent work improvements.

#### Inadequate supervision weakens expenditure performance.

The current focus of healthcare finance is on the compliance management of various healthcare project funds, tracking the progress of special fund expenditures, and the construction management of major projects, with insufficient attention paid to the effectiveness of fund use. Regulatory authorities lack attention to the social benefits generated by fiscal investments and improvements in healthcare service accessibility, placing excessive emphasis on the compliance of fund expenditures. Supervision is mostly ex post facto, and preventive, institutionalized long-term regulatory mechanisms are not sufficiently established. These regulatory deficiencies have led to inefficient use of funds, leaving considerable room for improvement in the efficiency of financial expenditure on healthcare in various cities and prefectures. They have also hindered local governments from adjusting the structure and scale of healthcare resource investment based on actual conditions, affecting the optimal allocation of financial resources, making it difficult to effectively alleviate regional disparities, and preventing the full realization of social benefits. At the same time, during healthcare field audits, some audit authorities focus too much on compliance audits and pay insufficient attention to efficiency audits, neglecting deep-rooted problems such as the imperfect operation mechanisms of grassroots healthcare facilities, the low efficiency of the use of key special funds, and the low utilization rate of medical equipment. This approach fails to effectively identify and solve the root causes of the low efficiency and downward trend of medical and health care spending in Hunan Province, affecting the effectiveness of improving the performance of financial expenditure on medical and healthcare.

#### The mechanism of auditing to enhance the performance of financial expenditure on medical and healthcare.

As an important part of national governance systems and governance mechanisms, government auditing effectively fulfills its audit function through its unique supervisory role, becoming a key tool for resolving the above issues and enhancing healthcare spending efficiency [51,52]. Especially in the context of low performance in medical and health expenditures and declining productivity, auditing plays an even more important role. According to the theoretical analysis and practical experience discussed above, auditing’s effect on healthcare expenditure performance is primarily reflected in three principal aspects:

#### Revealing problem mechanisms.

Through inspection and supervision of local government’s implementation of national health-related policies, illegal practices and irregularities in government healthcare financial expenditure are exposed by government audits in accordance with the law. At the same time, institutional mechanisms, institutional loopholes, and management deficiencies are revealed in the use of healthcare funds by governments at all levels. Audit information and recommendations are submitted to relevant government departments to promote effective improvements in these areas, thereby positively influencing the improvement of healthcare expenditure performance. Audit authorities regularly publish audit results and submit audit information and recommendations to relevant departments, providing authentic and objective information support to ensure the implementation of national healthcare policies. This can effectively reduce information asymmetry between the public and the government while guiding public opinion to supervise government departments through transparent information disclosure [53]. Under the dual effects of government audits and social supervision, risks in the use of healthcare funds can be effectively prevented, ensuring the maximization of public health investment returns and driving continuous improvements in healthcare expenditure performance across regions.

#### Enforcement and rectification mechanism.

In response to identified issues, audit authorities employ two key measures: enforcement and penalties, and the issuance of rectification recommendations. These measures drive audit accountability, facilitate the implementation of corrective actions, and encourage relevant government departments to actively implement healthcare policies. This ensures and promotes the judicious distribution and efficient utilization of healthcare resources while preventing power misuse that may harm public interests [54,55]. Audit authorities strictly enforce punitive measures, increasing the cost of non-compliance for audited entities and creating a unique and powerful deterrent effect against potential violations. The corrective action recommendation function involves conducting in-depth analyses of the root causes of identified issues, focusing on systemic deficiencies and management loopholes revealed by non-compliance issues. These findings are effectively integrated into systemic improvements, with recommendations for comprehensive corrective actions that address both symptoms and root causes, thereby ensuring the enhancement of healthcare expenditure performance.

#### Risk prevention mechanisms.

Audit agencies leverage their inherent deterrent effect and independent advantages to conduct multi-dimensional inspections and assessments of medical expenditures, comprehensively analyze the root causes of issues, optimize the management systems and mechanisms for financial expenditures in the healthcare sector, improve relevant expenditure regulations and systems, and effectively prevent and warn against potential risks in the implementation of healthcare policies [56]. This strong deterrent effect is more effective in preventing nascent issues from evolving into systemic problems and preventing localized issues from developing into widespread problems. Based on the issues identified and uncovered, audit work focuses on the weak links and potential risks in the use of healthcare funds, thereby correcting the alignment between local governments’ macroeconomic control objectives and the implementation outcomes of their decisions at critical junctures, and promptly urging adjustments and improvements to promote the implementation of national healthcare policies and measures.

To thoroughly investigate the logical connection between healthcare expenditure performance and subsequent incentives and mechanisms, and to provide data support for subsequent policy recommendations, this paper statistically analyzed the healthcare TE, audit capacity (AC), and audit disclosure performance (ADP) of each city in Hunan Province from 2014 to 2022, and performed correlation analysis on the data. Following the methodology of Li J et al. [57], Xing W and Gao Y [58], AC is measured by the number of audit personnel, as they form the core of audit resources and directly influence audit operations. Audit disclosure performance is assessed using the natural logarithm of the total amount of violations uncovered during audits. All audit-related data is sourced from the 2014–2022 China Audit Yearbook. As shown in Table 4, comparative analysis reveals:

**Table 4:** Correlation analysis of healthcare technology efficiency and audit-related indicators among cities in Hunan Province from 2014 to 2022.

	TE	AC	ADP
TE	1		
AC	0.390***	1	
ADP	0.263***	0.411***	1

Note: *** p < 0.01, **p < 0.05, * p < 0.1

According to the correlation analysis results, Table 2 shows the correlation coefficients between each variable. The analysis results show that there is a significant positive correlation between AC and TE, indicating that among cities in Hunan Province, areas with stronger audit staff allocations tend to have higher TE of healthcare expenditures. This statistical correlation is consistent with the logic that audit supervision promotes expenditure performance in the previous theoretical analysis. To a certain extent, this means that cities with higher TE of healthcare expenditures have stronger AC. Echoing the research findings of Zeng and Li [59] at the provincial level, the stronger the audit force, the stronger the audit execution, the greater the supervision of fiscal expenditures, and the more effectively it can improve fiscal expenditure performance. Therefore, the analysis results of this article at the city level and in the medical and health field provide data support for the role of auditing power in improving the performance of healthcare financial expenditures at the local level.

During the audit process, identifying and reporting violations of laws and regulations by the audited entity, the higher the number of major issues identified, the higher the audit disclosure performance [60]. The correlation coefficient between ADP and TE is 0.263, showing a significant positive correlation between the two. This shows that cities with larger amounts of problems found in audits usually show higher expenditure efficiency, which is consistent with the audit mechanism of “finding problems - supervising rectification - performance improvement” mentioned above, and provides empirical evidence of the practical effectiveness of this mechanism. Combining existing research and analysis at the city level in Hunan Province, the positive correlation between AC and ADP on medical and health TE has a strong theoretical basis and practical support. Therefore, it can be seen that enhancing audit resource allocation and improving audit performance can actively facilitate improvements in healthcare expenditure technical efficiency, and also provides preliminary evidence for the role of audits in enhancing healthcare financial spending performance. Given that the sample size of this study is relatively limited, and the research focus is on performance measurement, problem identification, and exploring the theoretical mechanisms and practical paths for auditing to promote performance improvement, correlation analysis can provide preliminary empirical support for the auditing mechanism and meet the needs of the current research stage.

## Audit recommendations for enhancing the performance of healthcare expenditure in Hunan Province

### Improve the design of performance audit indicators

A multi-dimensional indicator system should be established that not only includes common indicators such as system construction, goal setting, and fund implementation, but also takes into account the significant differences between cities and prefectures to set effectiveness indicators such as the operational efficiency of medical institutions, the rationality of medical resource allocation between regions, the coverage of basic public health services, and the accessibility of medical treatment for the masses, in order to comprehensively evaluate the overall performance of Hunan Province’s financial expenditure on medical and healthcare in terms of economic and social benefits. For areas such as Changsha with better resource allocation, focus should be on reviewing the stability of resource allocation and service quality, and setting indicators such as satisfaction with medical service quality and completion rate of key specialty construction to prevent resource waste and efficiency decline. For areas such as Xiangtan and Shaoyang, where there is a mismatch between investment scale and demand, the coverage of standardized construction of primary medical institutions should be reviewed for insufficient investment. Indicators such as bed utilization rate, annual frequency of use of high-end medical equipment, and idle resource activation rate will be reviewed for excessive investment. The lag in technological progress identified in empirical analyses that constrains total factor productivity growth, it is imperative to refine technology-related indicators. These should include the renewal rate of medical equipment, the proportion of investment in medical technology innovation, the coverage rate of medical personnel training, the effectiveness of process optimization, and the interoperability rate of information systems. This approach must focus not only on the “hard capabilities” of clinical technology but also emphasize the “soft support” provided by management and information technology, thereby establishing a comprehensive framework to guide technological advancement. Based on the differences between cities, regional coordinated development indicators should be set, including the ratio of the highest and lowest city per capita fiscal health expenditures, the growth rate of fiscal transfer payments in backward areas, and the completion rate of dispatching medical experts from developed areas to backward areas to narrow regional gaps. Through comprehensive audits, issues such as uneven resource allocation, low service efficiency, and slow technological progress in Hunan Province’s healthcare expenditures can be revealed, and targeted improvement recommendations can be proposed to avoid the drawbacks of the traditional system, which prioritizes investment over benefits and hardware construction over service quality.

### Deepening the advancement of big data auditing

Audit authorities should strengthen their big data thinking, enhance data collection and management, systematize fragmented information, and ensure that incompatible information can be effectively integrated. They should actively improve their big data auditing capabilities and expand the breadth and depth of auditing. Enhance the data collection and management systems within the healthcare sector across 14 cities in Hunan Province. The Provincial Department of Finance will take the lead, and the Provincial Health Commission, Medical Insurance Bureau, municipal finance bureaus, and health bureaus will participate together to unify data collection standards and interface specifications. Focus on collecting financial allocation data, health resource data, medical service data, and equipment asset data. Give priority to completing system docking in areas with better data foundations, such as Changsha, Zhuzhou, and Xiangtan, and gradually promote it to Zhangjiajie, Xiangxi, and other areas. Realize data interconnection and interoperability between medical and health departments at all levels and financial fund-using units, thereby building a data-based audit network and achieving comprehensive audit coverage in the true sense. Faced with massive amounts of data with low value density, audit agencies should speed up the construction of local fiscal expenditure performance audit information databases and build online audit platforms. With the help of data analysis tools such as Python, R, and GIS, and data mining and visualization technologies, they can conduct in-depth comparative analysis and trend predictions of medical and health performance indicator data from different sources. The focus should be on auditing the performance of healthcare financial expenditure, the effectiveness of healthcare reform policy implementation, and the optimisation of healthcare expenditure structures. This will help promptly identify deviations in policy implementation and issues related to fund usage efficiency, preventing public fund loss or wastage. Additionally, the long-term application of big data auditing will create a deterrent effect, strengthening preventive functions and encouraging all units to voluntarily standardize their use of healthcare financial funds, thereby improving the quality and efficiency of healthcare financial expenditure performance audits.

### Strengthen comprehensive audit supervision and management

Audit authorities should shift away from the traditional practice of focusing excessively on post-event supervision and compliance audits, instead moving the focus of supervision to earlier stages. They should implement comprehensive tracking supervision throughout the entire process, including pre-event, during-event, and post-event stages, covering all aspects of medical and health projects, such as project initiation, fund allocation, and usage management. Performance audits ought to be integrated into the budgeting and planning decision-making process to evaluate the scientific and rational nature of healthcare financial expenditure decisions made by the 14 municipal governments in Hunan Province. The review focused on assessing the appropriateness of investment scales for major projects and the attainability of projected performance targets. Empirical analysis indicates that most cities in Hunan Province exhibit increasing returns to scale. Audit departments should focus on the volume and allocation methods of healthcare resource investments to ensure reasonable funding for healthcare and other public welfare sectors, avoiding an excessive pursuit of short-term economic benefits at the expense of basic public service provision. During the project implementation phase, dynamic tracking and supervision should be continuously conducted for major public health projects, the construction of grassroots healthcare service systems, and the development of key specialties, while establishing a regular audit system and a hierarchical early warning mechanism. Focus on supervising the progress of fund allocation, equipment procurement compliance, and staffing implementation. Timely discovery and correcting problems such as uneven resource allocation and duplication of construction, to ensure that the project is efficiently advanced according to the established goals. After project completion, a thorough audit shall be conducted to comprehensively evaluate the effectiveness of public funds utilisation across multiple dimensions, including target attainment, input-output efficiency, and the role in driving technological advancement. The assessment shall prioritise identifying issues such as inefficient allocation of healthcare resources and sluggish technological progress. The evaluation results will be fed back into future planning decisions to form a virtuous cycle.

Throughout the entire process—before, during, and after—audit authorities should strengthen coordination and cooperation with departments such as health, finance, and medical insurance to achieve comprehensive and precise supervision of healthcare expenditures, form a supervisory synergy, ensure the safe, standardised, and efficient use of healthcare fiscal funds, and comprehensively improve the performance of healthcare financial expenditures.

### Strengthening the application of audit results

#### Enhance the audit result announcement system.

Audit result reports and audit announcement systems represent key vehicles for promoting audit information transparency. Hunan Province should focus on improving the medical and health financial expenditure performance audit result reporting and audit announcement systems, and strengthen the rectification of problems found in audits and the disclosure of results. Establish a hierarchical and classified announcement mechanism, and disclose the main problems discovered by audits and rectification requirements for areas with low resource allocation efficiency and inefficient use of fiscal funds. In particular, information concerning the use of funds for medical and health service projects, major equipment procurement, and the construction of grassroots medical institutions should be disclosed, such as the list of long-term idle or inefficiently used medical equipment and the responsible units, the list of medical institutions with significantly low bed utilization rates, and the gap in per capita financial health expenditures between cities and the implementation of transfer payments, and solicit opinions through government websites, new media, and other channels. This will prompt government departments to promptly rectify the audit problems discovered, and continuously supplement and improve relevant rules and regulations that support the development of medical and health finance, thus ensuring government credibility and financial information transparency.

#### Strengthen accountability for audit rectification.

Follow-up audit inspections mainly focus on the execution of audit recommendations and corrective actions. By providing timely feedback regarding the execution of audit recommendations and remedial measures taken by audited entities, the realization of health sector audit goals can be ensured. In response to the conclusions drawn in the empirical part, the audit department needs to track key indicators such as the amount of investment in the medical and health field, the proportion of investment in technological innovation, and the level of informatization, to enhance economies of scale, promote technological progress, and optimize the efficiency of fiscal fund utilization. Simultaneously, the working coordination mechanism between the Hunan Provincial Audit Authority and the finance department and budget department should be established and improved, using audit results and rectification status as important references for next year’s healthcare fiscal budget allocation. The effectiveness of audit supervision is not only reflected in identifying issues and ensuring the implementation of corrective measures but should also focus on whether those responsible for the issues have been appropriately disciplined to prevent similar issues from recurring and serve as a deterrent. Therefore, further utilize the results of healthcare sector audits to strengthen audit supervision, integrate policy tracking audits, leadership economic responsibility audits, and performance audits, and hold accountable units and individuals for violations of duties or inadequate fulfillment of responsibilities to maximize the utilization of audit outcomes.

## Conclusion

The findings reveal that healthcare financial expenditure performance levels in Hunan Province are generally modest, with most cities experiencing increasing returns to scale in healthcare expenditure. The Malmquist productivity index for healthcare financial expenditures has declined overall, primarily due to a low index of technological progress. The key determinants of weak performance include flaws in the fiscal expenditure system, inadequate performance evaluation, and insufficient oversight. The study has further confirmed the beneficial effect of auditing on healthcare financial expenditure performance. In provinces with higher technical efficiency, audit functions are more robust. Therefore, to improve the performance of healthcare financial expenditures, the key lies in improving the performance audit indicator system, innovating audit techniques and methods, perfecting audit supervision systems, and strengthening the application of audit outcomes. These measures are essential for resolving existing difficulties and fostering the sustainable and efficient development of healthcare financial expenditures.

Our study has several limitations. First, while the DEA-Malmquist model is employed to systematically evaluate the static efficiency and dynamic trends of healthcare financial expenditures, with a primary focus on measuring the relative efficiency of inputs and outputs, the current research phase does not incorporate the potential impacts of socioeconomic contextual variables on efficiency. The measured efficiency values cannot distinguish the effects of environmental constraints and management factors. It is not appropriate to simply attribute efficiency differences to the quality of management. Second, through cross-sectional data analysis, it is found that audit indicators and efficiency indicators are related, which provides preliminary support for the theoretical mechanism. However, cross-sectional data cannot establish the direction of causality. This correlation may be affected by reverse causation or common factors, and no mediating effect model has been constructed to test the effectiveness of the specific transmission path. Therefore, “strengthening audit supervision will help improve efficiency” should be understood as reasonable speculation based on theory and relevant evidence, rather than causal confirmation. Future research will use second-stage regression analysis to incorporate socioeconomic control variables such as population density, age structure, and disease burden, and use causal identification methods and mediating effect models on the basis of expanding the sample and improving variables to further test the causal effect and mechanism of audit on the performance of healthcare financial expenditures. In addition, this study found that economically underdeveloped regions such as Shaoyang and Yongzhou show diminishing RTS, which is inconsistent with traditional cognition. The underlying mechanisms behind it, such as transfer payment system design, local government decision-making bias, or service supply and demand mismatch, are worthy of in-depth exploration in the future.

## Supporting information

S1 Data Dataset. (XLSX)

## Abbreviations

SFA: Stochastic frontier analysis
DEA: Data envelopment analysis
CCR: Charne, Cooper, Rhodes
BCC: Banker, Charnes, Cooper
DMU: Decision-making unit
TFPCH: Total factor productivity change
EFFCH: Technical efficiency progress change
TECHCH: Technical progress change
PECH: Pure technical efficiency change
SECH: Scale efficiency change
TE: Technical efficiency
PTE: Pure technical efficiency
SE: Scale efficiency
RTS: Returns to scale
AC: Audit capacity
ADP: Audit disclosure performance.
